# Prostate cancer tissue mapping and stratification using DRAQ5 and Eosin fluorescent labels integrated with AI classification and segmentation algorithms

**DOI:** 10.1371/journal.pone.0345014

**Published:** 2026-03-26

**Authors:** Michail Georgios Papachristos, Emiliano Spezi, Carolina Fuentes, Ioulia Evangelou, David Hywel Thomas, Fiyinfoluwa Akinade, Marie Wiltshire, Anna Wilson, Rachel J. Errington, Dimitris Parthimos

**Affiliations:** 1 School of Medicine, Division of Cancer and Genetics, Cardiff University, Cardiff, United Kingdom; 2 School of Engineering, Cardiff University, Cardiff, United Kingdom; 3 School of Computer Science and Informatics, Cardiff University, Cardiff, United Kingdom; 4 Swansea Bay UHB Pathology Laboratories, Morriston, Singleton, Swansea, United Kingdom; 5 Department of Cellular Pathology, University Hospital of Wales, Heath Park, Cardiff, United Kingdom; 6 University of Alabama at Birmingham School of Medicine, Birmingham, Alabama, United States of America; Bayer Crop Science United States: Bayer CropScience LP, UNITED STATES OF AMERICA

## Abstract

**Background:**

Fluorescent microscopy using the DRAQ5 and Eosin probes has been shown in the literature to be capable of producing rapid tissue characterization through synthetic H&E-like pseudoimages, which can be potentially utilized in the clinic. This study focuses on developing deep learning models for classification and segmentation of prostate tissue labeled with DRAQ5&Eosin. The fluorophores provide highly specific features of nuclear and cytoplasmic content that allows for enhanced spatial resolution and multi-parametric analytics. The inter-dependencies of image acquisition and configuration variability on AI predictive accuracy is systematically interrogated. We are thus able to establish limits on experimental and analytical robustness in automated Gleason Grading (1–5) tissue samples of prostate cancer.

**Materials and methods:**

A labeling technique based on a far-red DNA probe DRAQ5, and Eosin allowed us to generate a two-channel fluorescent readout of prostatic tissue samples. Deep learning networks were employed to classify and segment DRAQ5 and Eosin fluorescent image regions into healthy and high/low grade cancerous tissue. A subset of images were acquired with variable microscopy configurations (focus, noise, zoom, lens) to evaluate the robustness of the proposed experimental-analytical pipeline and reproducibility of predictions.

**Results:**

Machine Leaning classifiers of High Grade Cancer (Gleason pattern 4 or 5) vs Healthy, Low Grade Cancer (Gleason pattern 3) vs Healthy, and High Grade Cancer vs Low Grade Cancer achieved an area under the curve of 0.9314, 0.8398, and 0.7715 respectively. Pixel wide cancer segmentation attained DICE scores of 0.8436, 0.5138, and 0.705 for background, healthy, and cancerous tissue respectively. The segmentation model also displayed robustness against a broad range of induced acquisition variability.

**Conclusion:**

Overall, DRAQ5 and Eosin labeling in combination with AI tools demonstrate a potential pipeline used in diagnostic clinical application when employing fluorescent imaging. Future research could expand and bring this combined fluorescent biomarker and AI methodology to the clinic.

## Introduction

Prostate cancer (PCa) is a disease that affects millions of people every year worldwide, with approximately 1,400,000 people being diagnosed with prostate cancer in the year 2022, and near 400,000 individuals dying because of the disease [1]. The clinical diagnosis pipeline for prostate cancer commonly involves prostate-specific antigen (PSA) level measured in blood serum, in combination with digital rectal examination (DRE). In the UK, PSA is a very commonly examined biomarker, especially for middle aged men between 45 and 69 years old, with a proportion of PSA tests being done for prostate cancer [2]. Based on the combination of the findings from the PSA level measurement and DRE, the pathologist assesses the risk for prostate cancer, and if recommended a prostate biopsy is performed. Tissue obtained via biopsy is commonly stained by a protocol involving a combination of Hematoxylin and Eosin (H&E). The standard way to quantify the stage and progression of prostate cancer is through Gleason Grading of H&E stained prostate tissue [3]. Gleason Grading refers to the system used by pathologists to quantify prostate cancer progression based on the spatial patterns and architecture of the tissue [3].

**Fig 1:**
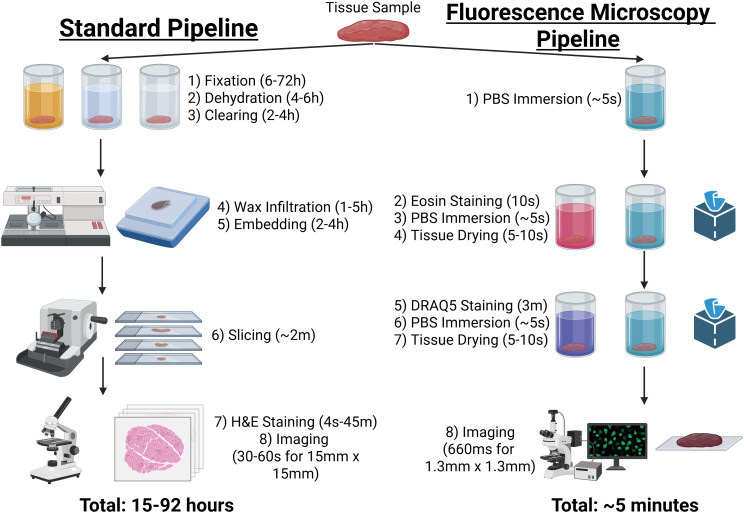
Schematic of tissue processing methodology. The standard pipeline for processing and staining tissue for a clinical setting is compared to the rapid real-time DRAQ5&Eosin staining fluorescent microscopy method by Elfer (2016) [9]. The individual steps and times required are presented for both methods. Information about the standard clinical method was retrieved from Suvarna et al. [15] (2018), with further information about the slicing step acquired from Buesa et al. [16] (2010). Time units are given (ms: milliseconds, s: seconds, m: minutes, h: hours). Created in BioRender. Papachristos, M. G. (2026) https://BioRender.com/li1anrz.

In the field of histopathology, there is a need for rapid and real time biopsy results due to conventional tissue processing taking nearly 24 hours [4]. One potential methodology developed for accelerated real time analysis is done using fluorescent microscopy (FM) imaging through fluorescent nuclear staining of the tissue [5–7]. A major advantage of FM is that the processing time for the tissue is greatly minimized compared to conventional H&E (approximately 5–10 minutes) [6,8,9]. Furthermore, FM allows for greater preservation of tissue characteristics [10], 3D imaging and reconstruction of the tissue without cutting/sectioning [6,11], and for digitization of the images (digital pathology) [5,6]. Moreover, FM images can be converted to digital realistic synthetic H&E images, which means pathologists would not require additional training to annotate them [9,12]. There have been multiple papers researching potential applications of FM technology on urology and prostate cancer real time imaging [5,12]. A recently developed fluorescent microscopy approach involves imaging of the tissue using the fluorescent nuclear stain DRAQ5 [13] in combination with Eosin [9]. This methods yields two separate fluorescent images from the tissue, one DRAQ5 image containing nuclear information, and another Eosin image containing cytoplasmic information (DRAQ5&Eosin) [9]. This allows for rapid imaging of fresh tissue at different depths without the need to freeze or cut the tissue [9]. DRAQ5 also has a very high specificity for DNA, compared to acridine orange [14]. Fig 1 compares the different steps and times required for the standard tissue processing pipeline compared to rapid DRAQ5&Eosin fluorescent staining of the tissue biopsy according to the Elfer et al. (2016) proposed methodology [9]. This method allows for the retrieval of virtual slices from the tissue without the need for slicing [9]. The standard tissue processing time can range between 15–92 hours, while the Elfer et al. pipeline can be done in approximately 5 minutes [9].

Over the years, there have been multiple Machine Learning (ML) tools applied to histological images of prostate cancer for classification and diagnostic purposes [17–20]. The potential benefits of the utilization of AI in the clinical environment have been recently shown [21] as AI-based algorithms for prostate cancer detection are being considered for widespread clinical use [22]. However, even though there is extensive research related to applying AI analysis of H&E prostate cancer tissue for diagnostic purposes, there seems to be a gap when it comes to literature concerning application of those same AI methods on fluorescent biomarker tissue. With FM being researched for use in the clinic [10], the next step of research and application would involve using AI in combination with FM for assisting in diagnostic purposes and accelerating the whole process.

The aim of this study is the development of an image pre-processing and AI pipeline on DRAQ5&Eosin (D&E) fluorescent image data for prostate cancer detection and segmentation purposes. A further focus of this study is a standardization analysis, which entails the validation of the constructed AI pipeline on D&E images acquired using multiple microscopy configurations. This is done to ensure the AI pipeline is robust and reproducible to different levels of acquisition variability.

## Materials and methods

### Overview of research pipeline and datasets

**Fig 2:**
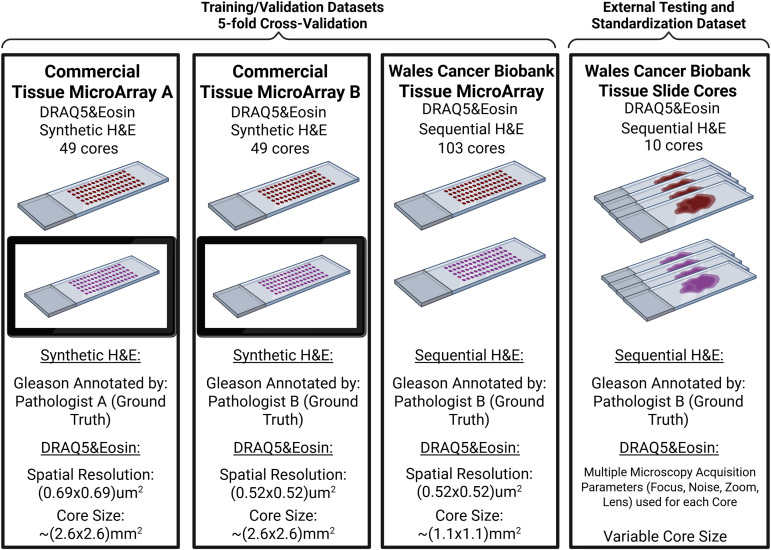
Schematic of datasets. The Commercial TMAs consisted of 49 needle biopsy prostate cores (samples from the two TMAs are the same but the slices are not sequential). The WCB TMA consisted of 103 cores. Commercial TMAs A and B along with the WCB TMA were all used as training/validation datasets (with 5-fold cross validation across the TMA cores). The WCB Tissue Slide Cores were used as the external testing and standardization dataset. Created in BioRender. Papachristos, M. (2026) https://BioRender.com/efd1l3q.

**Fig 3:**
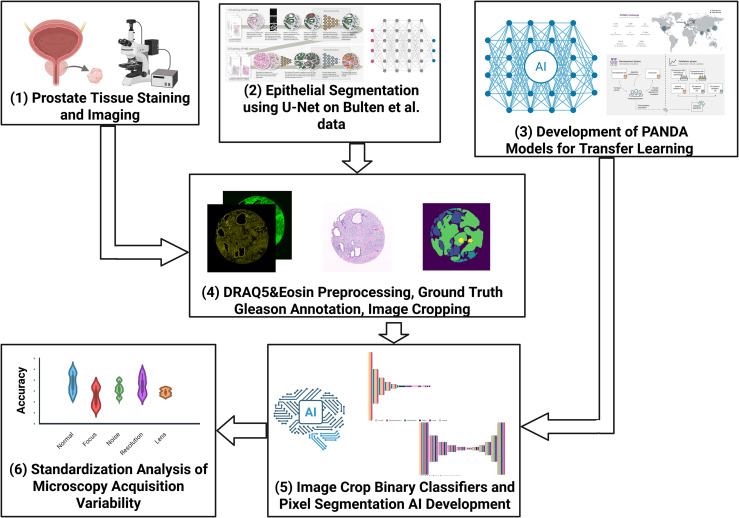
Schematic of research pipeline. The overall pipeline involves: (1) Prostate Tissue being stained with DRAQ5 and Eosin, and imaged through the ZEISS LSM 880 AxioObserver fluorescent microscope. (2) Epithelial Segmentation of the DRAQ5&Eosin images based on a U-Net trained on data provided by Bulten et al. [24]. Image adapted from Bulten et al. [24] (Creative Commons license: http://creativecommons.org/licenses/by/4.0/) (3) Development of AI models on the PANDA dataset [25] for transfer learning. Image adapted from Bulten et al. [25] (Creative Commons license: http://creativecommons.org/licenses/by/4.0/). (4) DRAQ5&Eosin preprocessing, Gleason annotation by expert pathologists, and image cropping. (5) Development of image crop binary classifiers (Healthy vs Low Grade Cancer, Healthy vs High Grade Cancer, Low Grade vs High Grade Cancer) and pixel cancer epithelial segmentation AI algorithms. (6) Standardization Analysis for AI robustness on images with variable microscopy configurations (focus, noise, sampling density, lens). Created in BioRender. Papachristos, M. (2026) https://BioRender.com/1s34cb8.

The datasets used in this study were a combination of two Commercial Tissue Microarrays (TMAs), one TMA obtained from the Wales Cancer Biobank (WCB) [23] and 10 additional cores also provided from the WCB [23]. The Wales Cancer Biobank is approved as a Research Tissue Bank by Wales Research Ethics Committee (REC) 3 and is licensed by the Human Tissue Authority under the UK Human Tissue Act (2004) to store human tissue for research (research licence 12107). The data from the Wales Cancer Biobank was accessed for research purposes on 17/01/2024. The Wales Cancer Biobank data was anonymized and contained no information that would allow for the identification of the identity of the participants. A detailed breakdown of the datasets is provided in Fig 2. The overall pipeline of the study involves the D&E staining and imaging of the tissue, followed by building models of epithelial segmentation using data by Bulten et al. [24], and utilizing The PANDA dataset [25] to generate models for transfer learning. The retrieved D&E images were preprocessed, the epithelial tissue was segmented based on the Bulten et al. [24] model built, and the ground truth was defined based on the epithelial mask generated combined with the pathologist annotations. Finally, the D&E models were constructed through transfer learning of the previously developed PANDA models, and validation along with standardization analysis was applied. The described steps of the overall pipeline are presented in detail in the following sections and visualized in Fig 3.

### Staining protocol

The staining was performed in a fume cabinet and involved the following steps:

1) Slides were dried in an oven for 1 hour at 60 °C to remove excess paraffin wax and water.
2) Slides were immersed in Xylenes (247642, obtained from Sigma-Aldrich) for 5 minutes (2 changes).
3) Slides were transferred to absolute Ethanol for 20 seconds (2 changes).
4) Slides were sequentially re-hydrated in 90%, 70% and 50% Ethanol for 20 seconds each, then rinsed in running tap water for 5 minutes.
5) Excess water was removed from around the sample and a Dako PAP pen was used to draw a delimiting well around the location of the tissue on the slide.
6) 500–750 ul of 0.5% Eosin Y (HT110216; 0.5% w/v in water, obtained from Sigma-Aldrich) was carefully pipetted on to the tissue, stained for 30 seconds and then the excess stain was quickly removed.
7) The slides were immersed in phosphate buffer solution (PBS) to remove excess stain and carefully dried off around the sample with paper towel.
8) 500–750 ul of 50 uM of DRAQ5 (5mM, DR5022, obtained from Biostatus) diluted 1:100 in PBS was overlaid onto the tissue, stained for 3 minutes and then the excess stain quickly removed.
9) Slides were rinsed with PBS.
10) 1–2 drops of Prolong Glass mounting medium (P36980, 2 mL, obtained from ThermoFisher Scientific) was applied to each tissue section and covered with a coverslip (thickness no. 1.5).
11) Samples were left for 24 hours at room temperature in the dark to dry before being imaged.

### Imaging protocol

This imaging protocol was developed for image acquisition of “Commercial TissueMicroarray A” previously imaged at the Tissue Microenvironment Group Labs. The ZEISS LSM 880 AxioObserver confocal laser scanning microscope (ZEISS, Oberkochen, Germany) was used to acquire the fluorescent images. The DRAQ5 and Eosin images were imaged at the same time. The objective lens used was the Plan-Apochromat 20x/0.8 M27 corresponding to a 20x magnification, 0.8 numerical aperture, a FWD of 0.55 mm, and a scan zoom of 0.6. Each pixel consisted of a (0.69×0.69)um^2^ area and had a bit depth of 12 bits. 8X confocal averaging was applied for denoising purposes. The same protocol was used for the image acquisition of the “Commercial TissueMicroarray B” and “WCB TissueMicroArray” with the only difference being the zoom was set to 0.8 resulting in a pixel area size of (0.52×0.52)um^2^.

For the acquisition of the WCB external standardization dataset, the tissue cores were collected under different microscopy conditions. The conditions include different levels of focus, numbers of averaging, zoom, and lens. The Plan-Apochromat 20x/0.8 M27 lens (Magnification: 20x, NA: 0.8, FWD: 0.55 mm) was used with a scan zoom of 0.8 to retrieve an image with a pixel area size of (0.52m×0.52)um^2^. In order to collect images of varying blur/focus for each core, a z-stack of images at five different depths (across 10 um) was collected at −5, −2.5, 0, + 2.5, and +5 um distance from the tissue. Regarding variable noise collection (different SNRs (Signal to Noise ratios), where a high number of confocal averaging corresponds to high SNR, and low number of confocal averaging corresponds to low SNR), each of the core images was retrieved at 8X, 4X, 2X, and 1X respectively. Regarding variable sampling density collection, each of the core images were retrieved at different zooms of 0.6 and 1.2 resulting in pixel area size of (0.69×0.69)um^2^ and (0.34×0.34)um^2^ respectively. Finally, for each core an image was also collected using the Plan-Apochromat 10x/0.45 M27 lens (Magnification: 10x, NA: 0.45, FWD: 2.1 mm).

The laser line used for the DRAQ5 channel had a wavelength of 633 nm, and the laser line used for the Eosin channel had a wavelength of 514 nm. For the “Commercial TissueMicroarray A,” the detection wavelength was set to 661–759 nm and to 519–670 nm for the DRAQ5 channel and Eosin channel respectively. For the “Commercial TissueMicroarray B” and “WCB Tissue Slide Cores,” the detection wavelength was set to 661–759 nm and to 521–621 nm for the DRAQ5 channel and Eosin channel respectively. Finally, for the “WCB TissueMicroArray,” the detection wavelength was set to 661–759 nm and to 528–614 nm for the DRAQ5 channel and Eosin channel respectively. The detector type was a photomultiplier tube. The pinhole size setting was set to maximum.

### Computational tools

The following tools were used for the image analysis and machine learning pipelines developed. The Fiji software (ImageJ v2.16.0) was used for image background correction [26]. The Python programming language v.3.10.4 [27] was utilized through the Anaconda Navigator software v.4.10.3 [28] and the JupyterLab v.3.0.16/ Notebook v.6.4.0 interface [29]. The tensorflow v.2.19.0 package was used for construction and training of the AI models [30]. The ZEISS ZEN lite microscopy software was used for Gleason annotation purposes. The Wacom Center software along with a tablet was also used for annotation purposes.

For tasks requiring high computational power, speed, or memory, the Advanced Research Computing at Cardiff (ARCCA) servers were used which were provided by the Wales Supercomputer infrastructure. More specifically, Hawk, which is a high-performance computing cluster, was used. These servers include multiple high-level CPUs, GPUs, RAMs, and storage.

### Gleason annotation and labeling

The prostatic tissue was Gleason annotated by two expert pathologists (one for “Commercial TissueMicroarray A,” and the other for all the remaining TMAs and cores). Because pathologists are trained to Gleason annotate H&E images, the D&E images of the Commercial TMAs were converted to synthetic H&E images using a method based on Elfer et al. [9]. The difference between our method and the Elfer et al. method involved using gamma factors higher than 1 as opposed to lower than 1. For the tissue retrieved from the WCB [23], sequential H&E slides (also provided from WCB) were used for the Gleason annotation instead.

Using the ZEISS ZEN lite microscopy software, CZI (https://www.zeiss.com/microscopy/en/products/software/zeiss-zen/czi-image-file-format.html) images were annotated. The expert pathologists performed the annotation on a TIFF/PNG/CZI file (either Synthetic H&E or Sequential H&E), and then the annotations in the annotated file were manually transferred into the CZI file in the ZEISS ZEN lite microscopy software. The annotation involved 6 different categories: Healthy, Gleason pattern 3, Gleason pattern 4, Cribriform pattern, Glomeruloid pattern, and Gleason pattern 5. The masks of the annotations were extracted from the annotated CZI file using a custom python script.

### Epithelial segmentation

In this paper we utilized the H&E data from the Bulten et al. paper [24] along with immunohistochemistry data to construct an epithelial segmentation algorithm [24]. The dataset contains whole slides of H&E along with their respective epithelial masks. We used this data to construct an epithelial segmentation U-Net [31] algorithm. We selected randomly 25 whole slide images from the Bulten et al. [24] dataset for training purposes, and 5 whole slide images for testing purposes. To minimize memory usage, each whole slide was reduced to half its size (pixel area size from (0.48x0.48)um^2^ to (0.96x0.96)um^2^). Two hundred-and fifty random crops from each tissue were selected (with a size of 256x256 pixels which correspond to (245.76x245.76)um^2^. Each of the collected crops was luminosity standardized and Vahadane stain normalized [32] (based on one training crop as normalization target) using the staintools python package (Github Repository: https://github.com/Peter554/StainTools). The multiprocess v.0.70.18 python package [33,34] was used during the stain normalization to reduce computational time. Each crop was also afterwards contrast limited adaptive histogram equalized using the scikit-image v.0.22.0 python package [35] and then divided by 255 to set the range between 0 and 1.

The architecture of the U-Net [31] consisted of a contraction path which contained four contraction blocks and three expansion blocks. Details about the U-Net model architecture are given in S1 Fig in the Supplementary Materials (figure created using the keras-visualizer package: https://github.com/mahyar-amiri/keras-visualizer). The image input size was set to (256, 256, 3). The weight kernel initializer for each layer with weights was set to he normal [36]. The loss function used for the training of the model was the Dice function, the optimizer used was Adam with a learning rate of 0.001, and the batch size was set to 32. The training lasted for 30 epochs and the parameters of the model which produced the lowest loss from the testing dataset was selected. Overall, the H&E image crops along with their respective epithelial segmentations were used to train and validate the U-Net segmentation model.

The D&E images collected were converted to synthetic H&E images, using the method by Elfer et al. [9]. Using Multi Otsu thresholding [37] by the scikit-image package [35] (number of bins set to 255), the synthetic H&E image was segmented through two thresholds (low and high intensity), and the region of the image with intensity lower than the high intensity threshold was set as the tissue mask for tissue background segmentation. Each RGB array of the image was contrast limited adaptive histogram equalized using the scikit-image python package [35]. Afterwards, the synthetic H&E image was cropped into (245.52x245.52)um^2^ image crops (this corresponded to 356x356 pixels for “Commercial TissueMicroarray A,” and 471x471 pixels for the “Commercial TissueMicroarray B,” WCB TissueMicroarray, and WCB Tissue Slide Cores (where the z-stack was used)). Each crop was luminosity standardized and vahadane [32] stain normalized (based on the same normalization crop target as before but after it had been histogram equalized [35]) using the staintools python package (Github Repository: https://github.com/Peter554/StainTools) and then divided by 255 for scaling purposes. Finally, each crop was resized using nearest-neighbor interpolation from the opencv v.4.11.0 python package [38] to (256, 256, 3) pixels, annotated using the trained segmentation U-Net, and resized back to its original dimensions. This resulted in an epithelial mask generated for each D&E image. Regions of the epithelial mask that fell outside the tissue mask were automatically set as background to remove false artifacts. It should be mentioned, that for the WCB Tissue Slide Cores dataset, the sharpest image across the z-stack for each tile was used for generating the epithelial masks. Details for determining the sharpest image across the z-stack in a tile are given in the Standardization paragraph.

### DRAQ5&Eosin image pre-processing

Both D&E images were background corrected through the rolling ball background subtraction algorithm of the Fiji software [26] with a rolling ball radius of 50 pixels using a sliding paraboloid. The epithelial mask generated before was overlayed with the pathologist’s Gleason annotation. Any region in the pathologist’s annotation not overlayed with epithelial mask was set to background. The DRAQ5 and Eosin images were contrast limited adaptive histogram equalized using the scikit-image python package [35] to account for the intensity acquisition microscopy variability presented across the tissue cores.

Afterwards, each D&E (background corrected) image was cropped into approximately (245.52x245.52)um^2^ image crops (this corresponded to 356x356 pixels for “Commercial TissueMicroarray A,” and 471x471 pixels for the “Commercial TissueMicroarray B” and WCB TissueMicroarray). Each individual image crop was assigned to a ground truth class (Healthy, Gleason pattern 3, Gleason pattern 4, Cribriform pattern, Glomeruloid pattern, and Gleason pattern 5) based on the most predominant class in the pathologist’s annotation within that crop (excluding non annotated regions). If a crop did not contain any of the pathologist’s annotation, meaning it only consisted of non-annotated regions, then that crop was removed from the dataset. The crop was also removed if the most predominant class (excluding background) contained less than 15% of the pixels of the whole crop, or if the most predominant class was less than 75% of all the classes contained (excluding background). This was done to remove image crops that contain mainly background or to avoid potentially noisy classification of the crop. Each crop’s D&E individual channels were resized using nearest-neighbor interpolation to 256x256 pixels and then z-normalized in order to account for the intensity variance across different fluorescent images.

### Image cross-validation methodology

The image crops of the training/validation datasets were separated into folds based on the cores/patients they belonged to, such that all crops from the same core and patient are kept in the same fold to avoid patient-level leakage. This was done through 5-fold cross validation consisting of image crops with 25 images per fold (two images left out). The five folds were randomly constructed through 1000 random permutations and selecting the fold configuration where the 2 classes in a given binary classification were most balanced. Using the following methods: for each fold, the ratio of number of image crops of class 0 to the number of image crops of class 1 across all the images was calculated. The mean squared error between the 5 ratios for each fold and an array of 1 with length 5 was determined to quantify how balanced the images in a random 5-fold cross validation were. The 5-fold cross validation that provided the smallest mean squared error was chosen. The left out images were added to the fifth fold. For each split, four of the folds were used for training the models, and one for tuning/validation in order to select the epoch where the model was optimal.

### PANDA dataset transfer learning

The deep learning models were initially trained with the PANDA dataset [25] containing more than 10,000 H&E PCa slides with Gleason labels, before being applied to the D&E dataset through transfer learning. This was due to the limited availability of D&E training data. This was done by converting the PANDA H&E images to synthetic D&E images. When it came preprocessing the PANDA dataset [25], 241 random whole slides were selected which were not Healthy (had a Gleason score) and were provided from Radboud (since they contained Gleason annotations). One hundred-and ninety-three were used as a training dataset, and 48 were used as a validation dataset. 50 random crops of 512x512 pixels corresponding to (245.76x245.76)um^2^ were retrieved from each grade contained in the tissue slide. A grade was considered part of the slide if at least 5% of the total epithelial tissue in it contained that grade. A crop was removed if the most predominant class contained less than 15% of the pixels of the whole crop, or if the most predominant class was less than 75% of all the classes contained (excluding background and stroma). Afterwards, the H&E crops were resized using nearest-neighbor interpolation to 256x256 pixels and converted to synthetic DRAQ5&Eosin images. For the creation of the DRAQ5 synthetic image, the 8-bit RGB H&E slide was converted to grayscale using the opencv v.4.11.0.86 [38] package and had Multi Otsu thresholding [37] applied to it with 2 thresholds using the scikit-image [35] package. Intensities below the lower threshold were defined as the nuclei mask. A binary erosion using scikit-image [35] was applied to that nuclei mask, and regions outside of that mask were set to 0 to keep only the nuclei in the image. The grayscale DRAQ5 image was reversed to convert the brightest to darkest regions (0 stays as 0 after reversal). Random gaussian noise was added to the grayscale image with a variance of 0.0001 followed by a morphology dilation and gaussian filtering with a sigma = 0.5, all done using scikit-image [35]. The synthetic DRAQ5 image was multiplied by 4095 to match a 12bit image and then z-normalized. For the creation of the synthetic Eosin image, the nuclei regions from the mask were set to 0 and then the H&E image was converted to grayscale and reversed to convert the brightest to darkest regions. Random gaussian noise was added to the grayscale image with a variance of 0.0001 followed by a morphology dilation and gaussian filtering with a sigma = 0.5, all done using scikit-image [35]. The synthetic Eosin image was multiplied by 4095 to match a 12bit image and then z-normalized.

### Deep learning neural network

For classification purposes, we classified Gleason pattern 3 cases as “Low Grade Gleason” and both Gleason pattern 4 (including cribriform and glomeruloid pattern) and 5 cases as “High Grade Gleason.” Three different binary classification models were constructed: Low Grade Cancer (Gleason pattern 3) vs Healthy, High Grade Cancer (Gleason pattern 4, Cribriform pattern, Glomeruloid pattern, and Gleason pattern 5) vs Healthy, High Grade Cancer vs Low Grade Cancer. Additionally, a cancerous epithelium pixel-wide segmentation AI was also developed. For each binary model, the synthetic D&E image crops retrieved from PANDA along with their associated ground truths were used to construct a deep learning neural network for classification. The Convolutional Neural Network (CNN) accepted an image size of (256, 256, 2) pixels, meaning both the DRAQ5 and Eosin image were used as an input simultaneously. Details about the model architecture of the CNN are presented in S2 Fig in the Supplementary Materials. The model used the Xavier uniform initializer [39] on each layer with weights. The loss function used for the training of the model was the binary cross-entropy, the optimizer used was Adam with a learning rate of 0.0001, and the batch size was set to 32. The training lasted for 20 epochs and the parameters of the model which produced the lowest loss from the validation dataset were selected for the transfer learning.

For the construction of the pixel-wide cancer segmentation model, the Healthy and Diseased (Gleason pattern 3,4, or 5) D&E image crops were retrieved from PANDA, and the segmentation ground truth cancerous epithelial masks were defined by setting regions of background or stroma to zero, healthy epithelium to one, and Gleason pattern 3, 4, or 5 epithelium to two for each crop. The architecture of the U-Net segmentation model [31] was similar to the one used for the epithelial segmentation of the synthetic H&E images. The differences were the image input size being set to (256, 256, 2), there being in total six contraction blocks, and five expansion blocks. Details about the model architecture of the Cancer Segmentation U-Net are presented in S3 Fig in the Supplementary Materials. The loss function used for the training of the model was the sparse categorical cross-entropy function, the optimizer used was Adam with a learning rate of 0.02, and the batch size was set to 32. The training lasted for 20 epochs and the parameters of the model which produced the lowest loss from the validation dataset were selected for the transfer learning.

After the models were trained based on the synthetic D&E images from the PANDAS dataset, transfer learning was applied to train the actual D&E images.The cross-validation method of the actual D&E data is outlined in the Image Cross Validation Methodology section. During the binary classification training of the actual D&E data based on the PANDA trained models, the model architecture remained the same but the Adam learning rate was set to 0.00002 and the number of epochs to 20. The fold that produced the highest Cohen Kappa score was chosen for standardization. During the pixel-wide cancer segmentation training of the D&E data based on the PANDA trained models, the model architecture remained the same but the Adam learning rate was set to 0.01 and the number of epochs to 10. The fold that produced the highest sum of DICE scores for each class was chosen for standardization. A schematic of the U-Net model architecture used for epithelial segmentation is provided in S4 Fig in the Supplementary Materials (made using the visualkeras v.0.1.4 package [40]). A schematic of the CNN model architecture is provided in S5 Fig in the Supplementary Materials (made using the visualkeras package [40]). A schematic of the U-Net model architecture used for pixel-wide cancer segmentation is provided in S6 Fig in the Supplementary Materials (made using the visualkeras package [40]).

For training of all deep learning models on the actual D&E dataset, data augmentation was applied on the training dataset to increase model predictive performance, This included: 1000 random image crop flips (500 horizontally, 500 vertically), 1500 random image crop rotations (500 with 90 degree rotation, 500 with 180 degree rotation, 500 with 270 degree rotation) using the opencv package [38]. Furthermore, 500 random gaussian noise additions with variance randomly ranging from 0 to 0.5 for each image crop, and 500 random gaussian blurs of the images with a sigma value randomly ranging from 0 to 5 and then an addition of gaussian noise (same parameters as before) using the scikit-image package [35]. These 3500 image crop augmentations were mixed in with the training dataset for each of the five folds.

Computational Gaussian Noise Addition: $O=I+\frac{1}{\sqrt{2\pi \sigma^{2}}}e^{\frac{-I^{2}}{2\sigma^{2}}}$. Where I is the input image, O is the output image, and $\sigma^{2}$ is the variance randomly ranging from 0 to 0.5. Computational Gaussian Blur Convolution: $O=I⊛(\frac{1}{\sqrt{2\pi \sigma^{2}}}e^{\frac{-I^{2}}{2\sigma^{2}}})$. Where I is the input image, O is the output image, and σ is the standard deviation randomly ranging from 0 to 5.

The D&E images were also trained for classification on a ResNet50 model [41] in tensorflow [30] initiated with weights retrieved from ImageNet [42]. More specifically, a 2D Convolutional layer with 3 filters, a kernel size of (3, 3), same padding, ReLU activation function, and Xavier uniform weight initializer [39] was used to convert the input of (256, 256, 2) to (256, 256, 3) to be used on ResNet50 [41]. The layers of the ResNet50 were frozen with the exception of the last 7 layers which were trainable, and finally, a fully connected layer (initiated with Xavier uniform weight [39]) with a sigmoid function was added for binary classification. The loss was set to binary cross-entropy, the optimizer was Adam, and the number of epochs was set to 10. This was done to compare training the models using only a baseline ImageNet [42] weight initialization, compared to the transfer learning from the PANDA dataset using synthetic D&E images.

### Standardization

The models developed were experimentally validated on the standardization dataset. In regards to the z-stack focus standardization analysis, each of the image crops across the z-stack were ranked from 1 to 5 with 1 being the most focused and 5 being the most unfocused crop. The focus level of the crop was determined using the numpy v.1.26.4 package [43] to calculate the gradient values across the x- and y-axis of the image. Afterwards, the square root of the addition of the squared values of the x- and y-axis gradients were calculated and the average of them was taken. The tile with the highest average was given a sharpness rank of 1 and the lowest average tile a sharpness rank of 5 (sharpness of images in each crop was determined before histogram equalization was applied [35]). This was done because the sharpest tile across a z-stack will vary due to the heterogeneity in regards to the flatness of the tissue. For the Noise/SNR standardization, each crop was ranked from lowest to highest noise based on the confocal averaging parameter (high SNR corresponding to a confocal averaging of 8, and low SNR corresponding to a confocal averaging of 1). For the zoom sampling density and the lens standardization analysis, due to xy-grid mismatch between the image crops, the phase cross-correlation registration technique by scikit-image [35] was used to match the location of the image crops across different configurations.

In order to correct for local shifts between the epithelial masks and the tissue, which was caused by tile stitching misalignments during microscopy data acquisition when the zoom or lens is changed, the phase-cross correlation image registration method from the scikit-image [35] package was made use of for the sampling density and lens microscopy variability analysis. This was done locally between the DRAQ5 crop and epithelial mask before the feature extraction by applying local phase-cross correlations at different sliding regions of the image with size (64, 64) pixels, then (32, 32) pixels, and finally (16, 16) pixels.

## Results

### Staining protocol and imaging results

The staining of one slide with DRAQ5&Eosin took approximately 2 hours. Each slide also needs to be left in the dark overnight for approximately 24 hours. The imaging time of a region of tissue depends on the lens and the zoom sampling density used during the acquisition. Table 1 contains the time that is required to image one tile (1200x1200 pixels) based on the lens and zoom sampling density used with a confocal averaging of 8.

**Table 1:** Time required to image one tile (1200x1200 pixels) based on the lens and zoom sampling density used with a confocal averaging of 8.

Lens	20X/0.8 M27	20X/0.8 M27	20X/0.8 M27	10X/0.45 M27
Sampling Density	(0.69x0.69)um^2^	(0.52x0.52)um^2^	(0.35x0.35)um^2^	(0.52x0.52)um^2^
Tile Area	(706.56x706.56)um^2^	(532.48x532.48)um^2^	(358.4x358.4)um^2^	(532.48x532.48)um^2^
Time	7.45s	5.06s	4.17s	3.28s

**Fig 4:**
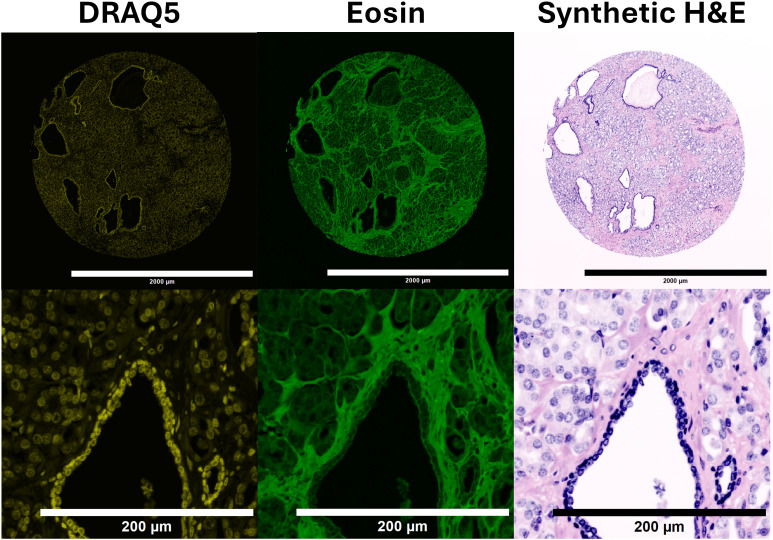
DRAQ5 and Eosin fluorescent images and corresponding synthetic H&E image. Top Row: Images. Bottom Row: Image crops of corresponding images from above.

**Fig 5:**
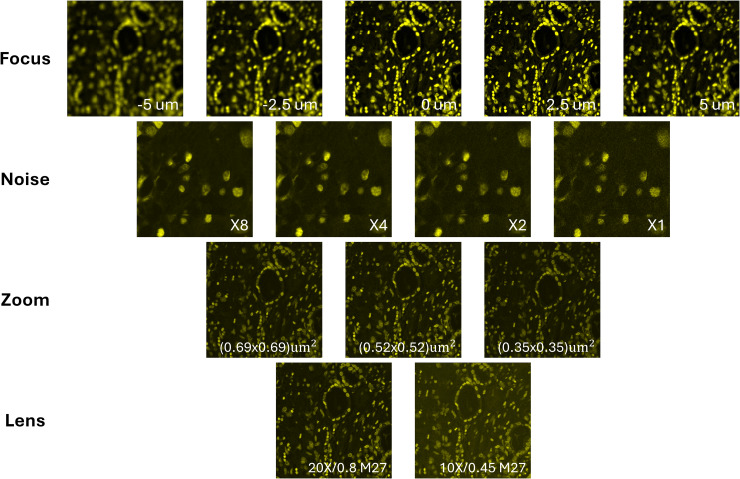
DRAQ5 images under different microscopy configurations. These include a z-stack for different levels of focus (−5, −2.5, 0, 2.5, 5 um), confocal averaging for different levels of noise (X8, X4, X2, X1), multiple zooms for different levels of sampling density (pixel area size of 0.69x0.69, 0.52x0.52, 0.35x0.35 um^2^), and two different lenses (20X/0.8 M27, 10X/0.45 M27).

**Fig 6:**
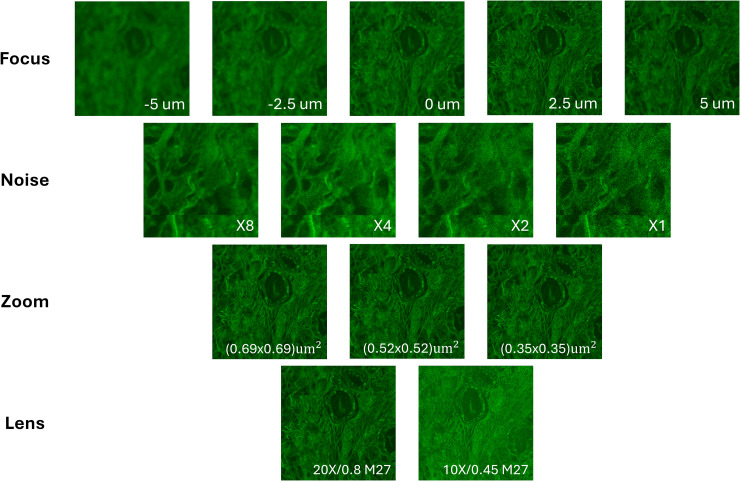
Eosin images under different microscopy configurations. These include a z-stack for different levels of focus (−5, −2.5, 0, 2.5, 5 um), confocal averaging for different levels of noise (X8, X4, X2, X1), multiple zooms for different levels of sampling density (pixel area size of 0.69x0.69, 0.52x0.52, 0.35x0.35 um^2^), and two different lenses (20X/0.8 M27, 10X/0.45 M27).

Fig 4 shows an example of DRAQ5 and Eosin fluorescent images along with the synthetic H&E. DRAQ5 and Eosin fluorescent image regions under different microscopy configurations are provided in Figs 5 and 6.

### Gleason annotation and epithelial segmentation results

Fourty six of the “Commercial Tissue MicroArray A” images were annotated by the pathologist, digitized, and used in the AI algorithm, including partially annotated images. Some images could not be annotated due to the need for immmunohistochemistry. Regarding the “Commercial Tissue MicroArray B,” all of the cores were annotated by the pathologist. Overall, 78 out of 103 WCB Tissue Microarray cores had a corresponding sequential H&E and Gleason annotation. For the WCB Tissue Slide, all cores were Gleason annotated.

**Fig 7:**
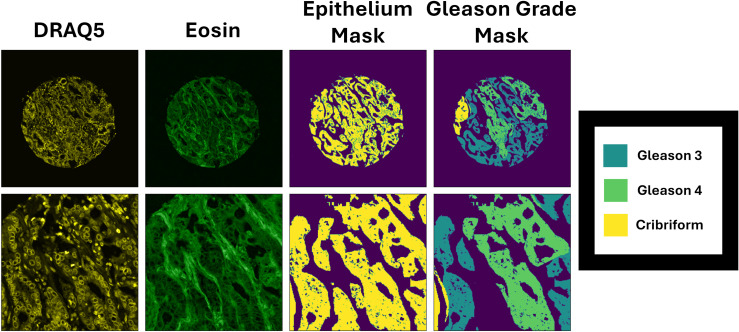
Epithelium mask, and Gleason grade mask of a DRAQ5&Eosin image. Top Row: Image. Bottom Row: Zoomed region of the image.

The epithelial segmentation model was able to achieve a Jaccard index of 0.7297 and a Dice score of 0.8437 on the validation dataset. An example of the Epithelium mask along with the Gleason annotation of a D&E image is presented in Fig 7.

The total number of Healthy, Low Grade (Gleason pattern 3), and High Grade (Gleason pattern 4 or 5) image crops from the Training/Validation Datasets, and the External Testing and Standardization Dataset are presented in Table 2.

**Table 2:** Total number of Healthy, Low Grade (Gleason pattern 3), and High Grade (Gleason pattern 4 or 5) image crops from the Training/Validation Datasets, and the External Testing and Standardization Dataset.

Type of Crop	Training/Validation Datasets	External Testing and Standardization Dataset
Healthy	1321	1045
Low Grade	1619	3608
High Grade	1590	1417

### PANDA models transfer learning results

With respect to the PANDA dataset, four different models were developed. Low Grade Cancer (Gleason pattern 3) vs Healthy classification, High Grade Cancer (Gleason pattern 4 or 5) vs Healthy classification, High Grade Cancer vs Low Grade Cancer classification, and cancerous epithelium pixel-wide segmentation. The Low Grade vs Healthy classification model was able to achieve an accuracy of 79.52%, Cohen Kappa score of 59.21%, and an AUC of 0.87. The High Grade vs Healthy classification model was able to achieve an accuracy of 84.03%, Cohen Kappa score of 66.39%, and an AUC of 0.92. The High Grade vs Low Grade classification model was able to achieve an accuracy of 79.98%, Cohen Kappa score of 56.16%, and an AUC of 0.88. The cancerous epithelium pixel-wide segmentation model was able to achieve a DICE Score of 0.9125 for background/stroma, 0.6502 for healthy tissue, and 0.8059 for cancerous tissue. In S7 Fig in the Supplementary materials, an example of an H&E region image along with the produced synthetic DRAQ5 and Eosin images in the PANDA dataset is displayed. In S8 Fig in the Supplementary materials, the ROC Curves, Loss Curves, and Accuracy curves are presented for each of the 4 models developed.

### Deep learning and standardization results

#### Training/validation data machine learning results.

**Fig 8:**
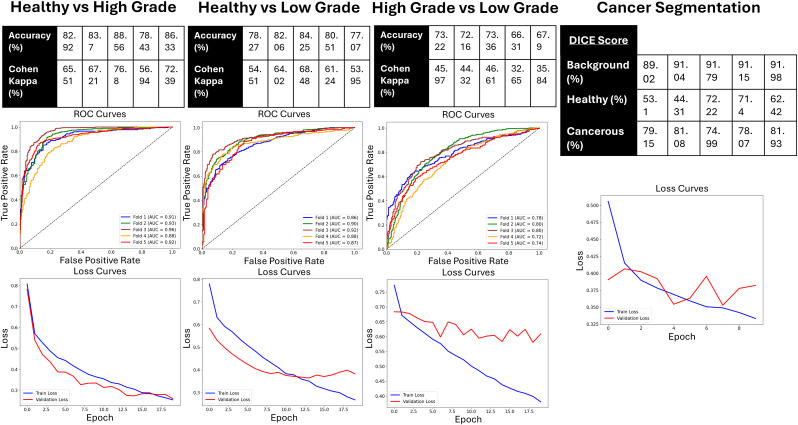
Training/validation data machine learning results. Figure contains Accuracies, Cohen Kappa Scores, ROCs, and AUCs of classification models for each fold, and Loss Curves of most predictive model of five folds for classification models. Figure also contains DICE Scores of Background, Healthy, and Cancerous Tissue for each fold, and Loss Curve of most predictive of five folds for segmentation models.

Fig 8 presents the Accuracies, Cohen Kappa scores and ROC Curves of the five models from each fold, and the Loss curve of the most predictive fold (based on the Cohen Kappa score), for the Low Grade Cancer vs Healthy, High Grade Cancer vs Healthy, and High Grade Cancer vs Low Grade Cancer classification models respectively. It also contains the DICE score for the background, healthy, and cancerous tissue of each fold and the Loss curve of the most predictive score (based on the sum of DICE scores).

Regarding the training/validation dataset, the highest predictive model was the Healthy vs High Grade with a max accuracy of 88.56%, Cohen Kappa of 76.8%, and an AUC of 0.96. The second most predictive was the Healthy vs Low Grade with a max accuracy of 84.25%, Cohen Kappa of 68.48%, and an AUC of 0.92. The least predictive model was the High Grade vs Low Grade model, with a max accuracy of 73.36%, Cohen Kappa of 46.61%, and an AUC of 0.8. The cancer segmentation model was able to achieve a DICE score of 0.9115 for background/stroma detection, 0.714 for healthy epithelium detection, and 0.7807 for cancerous epithelium detection.

#### External testing and standardization dataset results.

**Fig 9:**
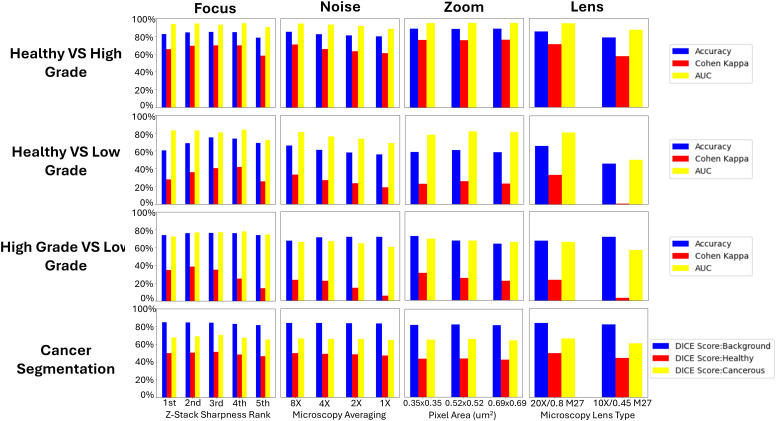
External testing and standardization dataset results. Figure contains Accuracies, Cohen Kappas, and AUCs for classification models across different microscopy conditions (sharpness rank, confocal microscopy averaging, sampling density pixel area, microscopy lens type) for standardization dataset. Figure also contains DICE Scores of Background, Healthy, and Cancerous for the segmentation model across different microscopy conditions.

Fig 9 contains boxplots of the accuracies, Cohen Kappa scores, and AUCs of the standardization dataset across different levels of focus, SNR, sampling density, and lens, for the Low Grade Cancer vs Healthy, High Grade Cancer vs Healthy, and High Grade Cancer vs Low Grade Cancer classification models respectively. It also contains boxplots of the background, healthy, and cancerous DICE score for the cancerous epithelium pixel-wide segmentation model across those same microscopy conditions. The time for running the model on the external testing dataset, across the four models, on one microscopy setting is approximately 30 minutes.

In the case of focus/unfocused microscopy configurations, the most predictive performance of the models for High Grade vs Healthy, Low Grade vs Healthy, and High Grade vs Low Grade were observed at the 3rd, 4th, and 2nd sharpness rank, with accuracies of 84.97%, 73.74%, and 76.36% respectively. The Cohen Kappa scores were 69.89%, 41.93%, and 38.59% respectively. The AUCs were 0.9314, 0.8398, and 0.7715 respectively. Overall the predictive performance from highest to lowest sharpness displays an initial increase until reaching an optimum, and then a decrease. The High vs Low grade model displayed the highest degree of sudden decrease.

Regarding noise, the predictive performance decreases along with the SNR of the images. The highest predictive power with an AUC from 0.9436 to 0.8857 is shown at the High Grade vs Healthy model, while the lowest predictive power with an AUC from 0.6605 to 0.6032 is shown at the High Grade vs Low Grade model. The predictive performance of the models remained consistent overall for the three different sampling densities used. Finally, changing the microscope lens from a high NA and 20X magnification lens to low NA and 10X magnification lens had the biggest effect on predictive performance. This was especially apparent for the Healthy vs Low Grade and High Grade vs Low Grade models where the AUC decreased from 0.8109 to 0.5020 and from 0.6605 to 0.5695 respectively.

**Fig 10:**
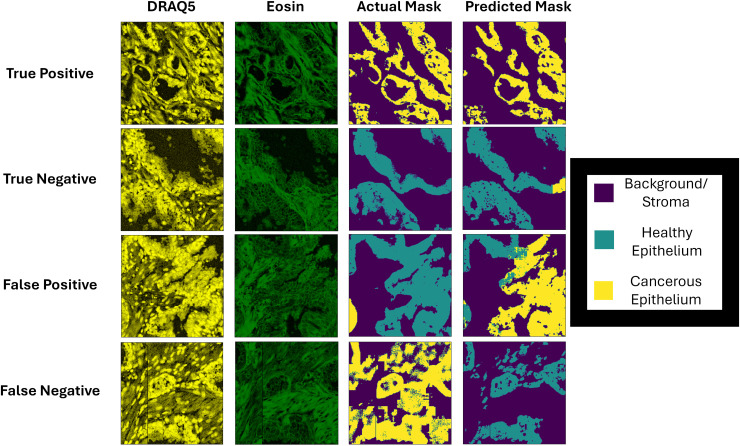
Examples of true positive, true negative, false positive, and false negative image segmentation of DRAQ5&Eosin image regions (after histogram equalization).

Interestingly, when considering the cancer segmentation methodology, the model displayed remarkable consistency across all the microscopy conditions, including the microscopy lens. A DICE score of 0.8436, 0.5138, and 0.705 for the background, healthy, and cancerous tissue was able to be achieved respectively on that external testing/standardization dataset. This suggests that the segmentation method is prone to false negatives, with false positives being rare. Fig 10 contains examples of D&E regions (after histogram equalization) along with actual and predicted masks from the segmentation model.

Table 3 contains the accuracies, Cohen Kappa scores, and AUCs of the classification models trained using transfer learning based on the synthetic D&E of the PANDA dataset compared to the classification models trained on the ImageNet dataset.

**Table 3:** Accuracies, Cohen Kappa scores, and AUCs of the classification models trained using PANDA compared to the classification models trained on ImageNet.

Classification	High Grade VS Healthy		Low Grade VS Healthy		High Grade VS Low Grade	
Training	PANDA	ImageNet	PANDA	ImageNet	PANDA	ImageNet
Accuracy	85.3%	76.4%	66.02%	60.93%	67.48%	62.97%
Cohen Kappa score	70.89%	52.42%	33.14%	27.56%	23.19%	24.59%
AUC	0.944	0.843	0.811	0.821	0.66	0.698

## Discussion

Overall, the models managed to achieve maximum accuracy of 84.97% with an AUC of 0.9314 for distinguishing between Healthy and High Grade cancerous regions. Similar results have been observed by Ali et al. [17] where an H&E region segmentation model was developed and a benign vs cancerous classification accuracy of 90% was achieved. However, when distinguishing between Healthy vs Low Grade Cancer, and Low Grade Cancer VS High Grade Cancer, the predictive performance of the model is moderate, with a maximum accuracy 73.74% with an AUC of 0.8398, and of 76.36% with an AUC of 0.7715 respectively. In comparison, Garcia et al. also developed an AI model for classifying between benign and Gleason pattern 3 glands that reported a maximum accuracy of 88.3±2.6% with an 0.922±0.045 AUC [19]. This suggests that the current AI pipeline is successful at differentiating between healthy and high grade cancerous tissue, but less adapted at predicting different levels of cancer progression in the tissue, or distinguishing the subtle variations between benign and Gleason pattern 3 tissue regions. Additionally, models trained on weights initialized in PANDA through transfer learning displayed a higher predictability compared to the models trained on ImageNet [42]. The exception to this was the Low Grade cancer vs Healthy model, which was the lowest performing model overall. Overall, these results demonstrate the potential predictive performance of D&E fluorescent images as a biomarker. It should be mentioned that the external testing dataset was imbalanced with the healthy, low grade, and high grade regions having a ratio of approximately 1:3:1 crops respectively.

When the trained models were tested against different microscopy sharpness configurations, they were found to overall be robust across the 1st-4th levels, with the highest predictive performance being observed between the 2nd and 4th sharpness rank of the focus augmentation. These findings suggest that including a certain degree of out-of-focus images in the training set increases the model’s predictive power. This can be most likely attributed to the AI algorithm generalizing mapping performance over a broader dataset. Regarding different levels of SNR, the 8X and 4X were relatively stable, with decreasing predictive performance starting from 2X and below. The rate of predictive performance decline was lower for Healthy vs High Grade compared to the other models. The models remained robust across all three sampling density levels, although its possible that if a lower sampling density was used the model performance would start to degrade. It should be mentioned that robustness to changes in sampling density does not necessarily imply robustness to true optical resolution degradation. Interestingly, the microscope parameter with the highest effect on performance was the lens with lower magnification and NA translating to a rapid decrease in predictive performance. These findings could potentially translate in the clinical setting, with low NA images having a negative effect on Gleason grading accuracy by expert pathologists. Overall, this systematic variability analysis highlights the importance of training AI models on histology across multiple lenses to ensure robustness and reproducibility between different datasets.

Annotation by the pathologists for a portion of the training/validation datasets (Commercial TMAs) was performed on synthetic H&E images, as there were no matching H&E images. Both pathologists were confident that the synthetic images contained all the morphological information for accurate Gleason scoring. Even though the training dataset was annotated mostly by using synthetic H&E data, the external testing dataset (which the model was tested/validated on) was labeled based on actual H&E slides that were sequential to the D&E based tissue slides. The similarity between synthetic H&E and actual H&E images was quantified by analysing pathologists’ annotations and is shown in S1 Table. The Elfer et al study provides a more in-depth analysis by comparing D&E with actual H&E images of different types of tissue, where common distinct morphological characteristics were identified between the two modalities by expert pathologists [9].

This study is a proof of concept in the application of AI on fluorescent histological pseudo-images for potential diagnostic optimization. D&E staining and imaging can potentially be used for real time grading of prostate cancer biopsies by pathologists. In the present study, however, we utilized formalin-fixed paraffin embedded tissue cores/slices, and our D&E staining methodology was not real-time. Although the process was different from the Elfer et al. paper [9], the fluorescent images retrieved from the two methodologies are of the same fluorescent modality. To our knowledge, one other study by Bianchi et al. [44] has utilized a similar approach of combining FM and AI on prostate cancer, but that study only focused on the distinction between healthy and cancerous tissue and not on cancer stratification, pixel wide segmentation, or standardization.

Limitations of this study and other relevant publications highlight the considerable amount of time required for annotation by expert pathologists and potential inter-observer variability [45]. To minimize these effects, we have engaged the contribution of two senior pathologists with extensive PCa grading experience. In the future, more robust and optimized models for fluorescent biomarker cancer prediction could be utilized with much larger training/testing datasets. Moreover, the feasibility of FM trained models on real-time imaged D&E prostatic tissue could be examined in future studies. Ultimately, the proposed methodology could be applied beyond prostate cancer in cases such as breast, lung, and brain cancers, where rapid and real time biopsies would be useful.

In conclusion, the AI cancer detection models developed for DRAQ5&Eosin were able to achieve a high predictive performance for both classification and segmentation. Superior grading accuracy is contingent on utilizing larger datasets. Crucially, we were able to demonstrate that the proposed models were robust to microscopy acquired noise, focus, sampling density, and lens variability. The results obtained support the potential clinical applicability of AI in combination with FM imaging in the clinic.

## Supporting information

S1 Fig Details about U-Net architecture of epithelial segmentation model (figure created using the keras-visualizer package: https://github.com/mahyar-amiri/keras-visualizer). (TIFF)

S2 Fig Details about CNN architecture of image crop classification models (figure created using the keras-visualizer package: https://github.com/mahyar-amiri/keras-visualizer). (TIFF)

S3 Fig Details about U-Net architecture of cancer-segmentation model (figure created using the keras-visualizer package: https://github.com/mahyar-amiri/keras-visualizer). (TIFF)

S4 Fig Schematic of U-Net architecture of epithelial segmentation model. (TIFF)

S5 Fig Schematic of CNN architecture of image crop classification models. (TIFF)

S6 Fig Schematic of U-Net architecture of cancer-segmentation model. (TIFF)

S7 Fig Examples of a H&E image region and the synthetically generated DRAQ5&Eosin image. (TIFF)

S8 Fig ROC curves, AUC, loss and accuracy curves of the models developed from the PANDA dataset. (TIFF)

S1 Table Comparison of manually transferred annotations (transferred from the sequential H&E to the D&E images), to the annotations by the two expert pathologists on the corresponding synthetic H&E (which is created from the D&E) on a subset of 9 WCB TMA cores. The inter-observer metrics (retrieved after epithelial segmentation is applied to the masks) for comparing mask overlap are the DICE score (which compares background to annotated region), and the quadratic weighted Cohen Kappa score (which also considers grades assigned (Healthy, Low Grade Cancer, High Grade Cancer)). Manual Tr: Manually Transferred Annotations, Path A: Pathologist A, Path B: Pathologist B.
(PDF)

## References

[1] Bray F, Laversanne M, Sung H, Ferlay J, Siegel RL, Soerjomataram I. Global cancer statistics 2022: GLOBOCAN estimates of incidence and mortality worldwide for 36 cancers in 185 countries. CA: a cancer journal for clinicians. 2024;74(3):229–63. 38572751 10.3322/caac.21834

[2] Young GJ, Harrison S, Turner EL, Walsh EI, Oliver SE, Ben-Shlomo Y, et al. Prostate-specific antigen (PSA) testing of men in UK general practice: a 10-year longitudinal cohort study. BMJ Open. 2017;7(10):e017729. doi: 10.1136/bmjopen-2017-017729 29084797 PMC5665300

[3] Epstein JI, Allsbrook WC Jr, Amin MB, Egevad LL, ISUP Grading Committee. The 2005 International Society of Urological Pathology (ISUP) Consensus Conference on Gleason Grading of Prostatic Carcinoma. Am J Surg Pathol. 2005;29(9):1228–42. doi: 10.1097/01.pas.0000173646.99337.b1 16096414

[4] Sivapathasundharam B, Panja P, Sriram G, Saraswathi T. Comparison of three different methods of tissue processing. J Oral Maxillofac Pathol. 2007;11(1):15. doi: 10.4103/0973-029x.33958

[5] Rocco B, Cimadamore A, Sarchi L, Bonetti LR, Bertoni L, Azzoni P, et al. Current and future perspectives of digital microscopy with fluorescence confocal microscope for prostate tissue interpretation: a narrative review. Transl Androl Urol. 2021;10(3):1569–80. doi: 10.21037/tau-20-1237 33850791 PMC8039586

[6] Liu Y, Levenson RM, Jenkins MW. Slide over: advances in slide-free optical microscopy as drivers of diagnostic pathology. Am J Pathol. 2022;192(2):180–94. doi: 10.1016/j.ajpath.2021.10.010 34774514 PMC8883436

[7] Glaser AK, Reder NP, Chen Y, McCarty EF, Yin C, Wei L, et al. Light-sheet microscopy for slide-free non-destructive pathology of large clinical specimens. Nat Biomed Eng. 2017;1(7):0084. doi: 10.1038/s41551-017-0084 29750130 PMC5940348

[8] Puliatti S, Bertoni L, Pirola GM, Azzoni P, Bevilacqua L, Eissa A, et al. Ex vivo fluorescence confocal microscopy: the first application for real-time pathological examination of prostatic tissue. BJU Int. 2019;124(3):469–76. doi: 10.1111/bju.14754 30908852

[9] Elfer KN, Sholl AB, Wang M, Tulman DB, Mandava SH, Lee BR, et al. DRAQ5 and Eosin ('D&E’) as an Analog to Hematoxylin and Eosin for Rapid Fluorescence Histology of Fresh Tissues. PLoS One. 2016;11(10):e0165530. doi: 10.1371/journal.pone.0165530 27788264 PMC5082869

[10] Ragazzi M, Piana S, Longo C, Castagnetti F, Foroni M, Ferrari G, et al. Fluorescence confocal microscopy for pathologists. Mod Pathol. 2014;27(3):460–71. doi: 10.1038/modpathol.2013.158 24030744

[11] Zhang Z, Ibrahim M, Fu Y, Wu X, Ren F, Chen L. Application of laser scanning confocal microscopy in the soft tissue exquisite structure for 3D scan. Int J Burns Trauma. 2018;8(2):17–25. 29755838 PMC5943615

[12] Eissa A, Puliatti S, Rodriguez Peñaranda N, Resca S, Di Bari S, Vella J, et al. Current applications of ex-vivo fluorescent confocal microscope in urological practice: a systematic review of literature. Chin Clin Oncol. 2024;13(4):52. doi: 10.21037/cco-23-150 38769791

[13] Smith PJ, Wiltshire M, Davies S, Patterson LH, Hoy T. A novel cell permeant and far red-fluorescing DNA probe, DRAQ5, for blood cell discrimination by flow cytometry. J Immunol Methods. 1999;229(1–2):131–9. doi: 10.1016/s0022-1759(99)00116-7 10556697

[14] Ortner VK, Sahu A, Cordova M, Kose K, Aleissa S, Alessi-Fox C, et al. Exploring the utility of Deep Red Anthraquinone 5 for digital staining of ex vivo confocal micrographs of optically sectioned skin. J Biophotonics. 2021;14(4):e202000207. doi: 10.1002/jbio.202000207 33314673 PMC8274380

[15] Suvarna KS, Layton C, Bancroft JD. Bancroft’s theory and practice of histological techniques E-Book. Elsevier Health Sciences; 2018.

[16] Buesa RJ. Productivity standards for histology laboratories. Ann Diagn Pathol. 2010;14(2):107–24. doi: 10.1016/j.anndiagpath.2009.12.005 20227016

[17] Ali T, Masood K, Irfan M, Draz U, Nagra AA, Asif M, et al. Multistage segmentation of prostate cancer tissues using sample entropy texture analysis. Entropy (Basel). 2020;22(12):1370. doi: 10.3390/e22121370 33279915 PMC7761953

[18] Bulten W, Pinckaers H, van Boven H, Vink R, de Bel T, van Ginneken B. Automated gleason grading of prostate biopsies using deep learning. 2019. https://arxiv.org/abs/1907.07980 10.1016/S1470-2045(19)30739-9 31926805

[19] García G, Colomer A, Naranjo V. First-stage prostate cancer identification on histopathological images: hand-driven versus automatic learning. Entropy (Basel). 2019;21(4):356. doi: 10.3390/e21040356 33267070 PMC7514840

[20] Li W, Li J, Sarma KV, Ho KC, Shen S, Knudsen BS, et al. Path R-CNN for Prostate Cancer Diagnosis and Gleason Grading of Histological Images. IEEE Trans Med Imaging. 2019;38(4):945–54. doi: 10.1109/TMI.2018.2875868 30334752 PMC6497079

[21] Bulten W, Balkenhol M, Belinga J-JA, Brilhante A, Çakır A, Egevad L, et al. Artificial intelligence assistance significantly improves Gleason grading of prostate biopsies by pathologists. Mod Pathol. 2021;34(3):660–71. doi: 10.1038/s41379-020-0640-y 32759979 PMC7897578

[22] Santa-Rosario JC, Gustafson EA, Sanabria Bellassai DE, Gustafson PE, de Socarraz M. Validation and three years of clinical experience in using an artificial intelligence algorithm as a second read system for prostate cancer diagnosis-real-world experience. J Pathol Inform. 2024;15:100378. doi: 10.1016/j.jpi.2024.100378 38868487 PMC11166872

[23] Parry-Jones A, Spary LK. The Wales Cancer Bank (WCB). Open Journal of Bioresources. 2018;5. doi: 10.5334/ojb.46

[24] Bulten W, Bándi P, Hoven J, Loo R van de, Lotz J, Weiss N, et al. Epithelium segmentation using deep learning in H&E-stained prostate specimens with immunohistochemistry as reference standard. Sci Rep. 2019;9(1):864. doi: 10.1038/s41598-018-37257-4 30696866 PMC6351532

[25] Bulten W, Kartasalo K, Chen P-HC, Ström P, Pinckaers H, Nagpal K, et al. Artificial intelligence for diagnosis and Gleason grading of prostate cancer: the PANDA challenge. Nat Med. 2022;28(1):154–63. doi: 10.1038/s41591-021-01620-2 35027755 PMC8799467

[26] Schindelin J, Arganda-Carreras I, Frise E, Kaynig V, Longair M, Pietzsch T, et al. Fiji: an open-source platform for biological-image analysis. Nat Methods. 2012;9(7):676–82. doi: 10.1038/nmeth.2019 22743772 PMC3855844

[27] Van Rossum G, Drake FL. Python reference manual. Amsterdam: Centrum voor Wiskunde en Informatica; 1995.

[28] Inc A. Anaconda software distribution. Anaconda Documentation. 2020.

[29] Kluyver T, Ragan-Kelley B, Pérez F, Granger B, Bussonnier M, Frederic J. Jupyter Notebooks–a publishing format for reproducible computational workflows. Positioning and power in academic publishing: Players, agents and agendas. IOS press. 2016. p. 87–90.

[30] Abadi M, Agarwal A, Barham P, Brevdo E, Chen Z, Citro C, et al. TensorFlow: large-scale machine learning on heterogeneous systems. 2015.

[31] Ronneberger O, Fischer P, Brox T. U-net: Convolutional networks for biomedical image segmentation. In: International Conference on Medical Image Computing and Computer-Assisted Intervention. 2015. p. 234–41.

[32] Vahadane A, Peng T, Sethi A, Albarqouni S, Wang L, Baust M, et al. Structure-Preserving Color Normalization and Sparse Stain Separation for Histological Images. IEEE Trans Med Imaging. 2016;35(8):1962–71. doi: 10.1109/TMI.2016.2529665 27164577

[33] McKerns MM, Strand L, Sullivan T, Fang A, Aivazis MA. Building a framework for predictive science. 2012. https://arxiv.org/abs/1202.1056

[34] McKerns M, Aivazis M. Pathos: a framework for heterogeneous computing. 2010. https://uqfoundation.github.io/project/pathos

[35] van der Walt S, Schönberger JL, Nunez-Iglesias J, Boulogne F, Warner JD, Yager N, et al. scikit-image: image processing in Python. PeerJ. 2014;2:e453. doi: 10.7717/peerj.453 25024921 PMC4081273

[36] He K, Zhang X, Ren S, Sun J. Delving Deep into Rectifiers: Surpassing Human-Level Performance on ImageNet Classification. In: 2015 IEEE International Conference on Computer Vision (ICCV). 2015. p. 1026–34. 10.1109/iccv.2015.123

[37] Otsu N. A threshold selection method from gray-level histograms. Automatica. 1975;11(285–296):23–7.

[38] Bradski G. The OpenCV Library. Dr Dobb’s Journal of Software Tools. 2000.

[39] Glorot X, Bengio Y. Understanding the difficulty of training deep feedforward neural networks. In: Proceedings of the thirteenth international conference on artificial intelligence and statistics, 2010. p. 249–56.

[40] Gavrikov P, Patapati S. visualkeras. 2020. https://github.com/paulgavrikov/visualkeras

[41] He K, Zhang X, Ren S, Sun J. Deep Residual Learning for Image Recognition. In: 2016 IEEE Conference on Computer Vision and Pattern Recognition (CVPR). 2016. p. 770–8. 10.1109/cvpr.2016.90

[42] Deng J, Dong W, Socher R, Li LJ, Li K, Fei-Fei L. Imagenet: A large-scale hierarchical image database. In: 2009 IEEE conference on computer vision and pattern recognition, 2009. 248–55.

[43] Harris CR, Millman KJ, van der Walt SJ, Gommers R, Virtanen P, Cournapeau D, et al. Array programming with NumPy. Nature. 2020;585(7825):357–62. doi: 10.1038/s41586-020-2649-2 32939066 PMC7759461

[44] Bianchi G, Puliatti S, Rodriguez Peñaranda N, Micali S, Bertoni L, Reggiani Bonetti L, et al. Artificial intelligence evaluation of confocal microscope prostate images: our preliminary experience. Minerva Urol Nephrol. 2023;75(5):545–7. doi: 10.23736/S2724-6051.23.05538-6 37728490

[45] Flach RN, Willemse P-PM, Suelmann BBM, Deckers IAG, Jonges TN, van Dooijeweert C, et al. Significant Inter- and Intralaboratory Variation in Gleason Grading of Prostate Cancer: A Nationwide Study of 35,258 Patients in The Netherlands. Cancers (Basel). 2021;13(21):5378. doi: 10.3390/cancers13215378 34771542 PMC8582481

