# Supplementary figures and images for: Prostate cancer tissue mapping and stratification using DRAQ5 and Eosin fluorescent labels integrated with AI classification and segmentation algorithms
Source: PLoS One. 2026 Mar 26;21(3):e0345014. doi: 10.1371/journal.pone.0345014 (PMC13021167; doi:10.1371/journal.pone.0345014)

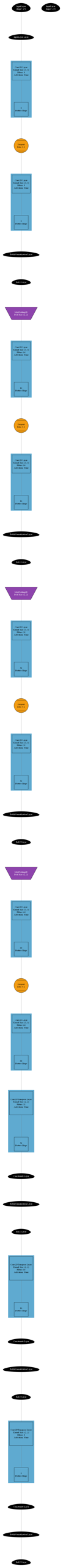

Supplement: S1 Fig — (TIFF) [file pone.0345014.s001.tiff]

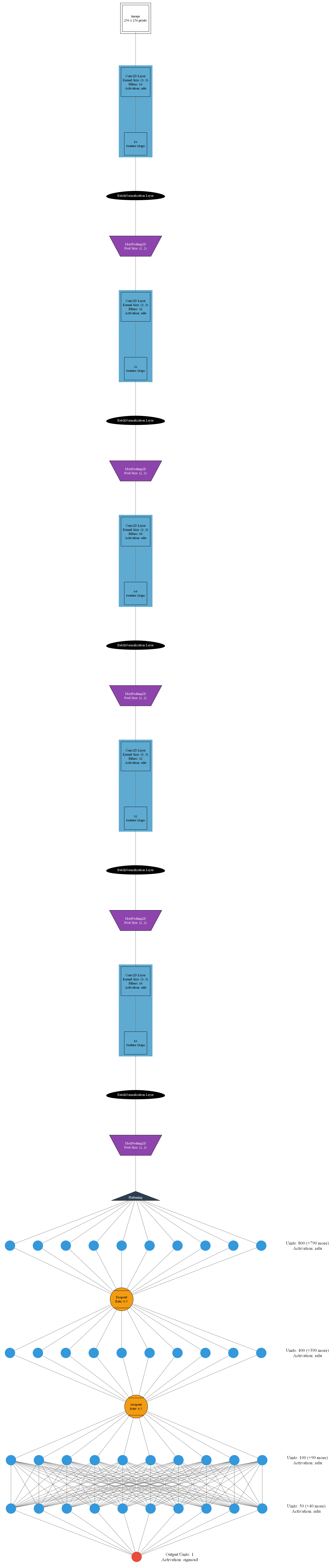

Supplement: S2 Fig — (TIFF) [file pone.0345014.s002.tiff]

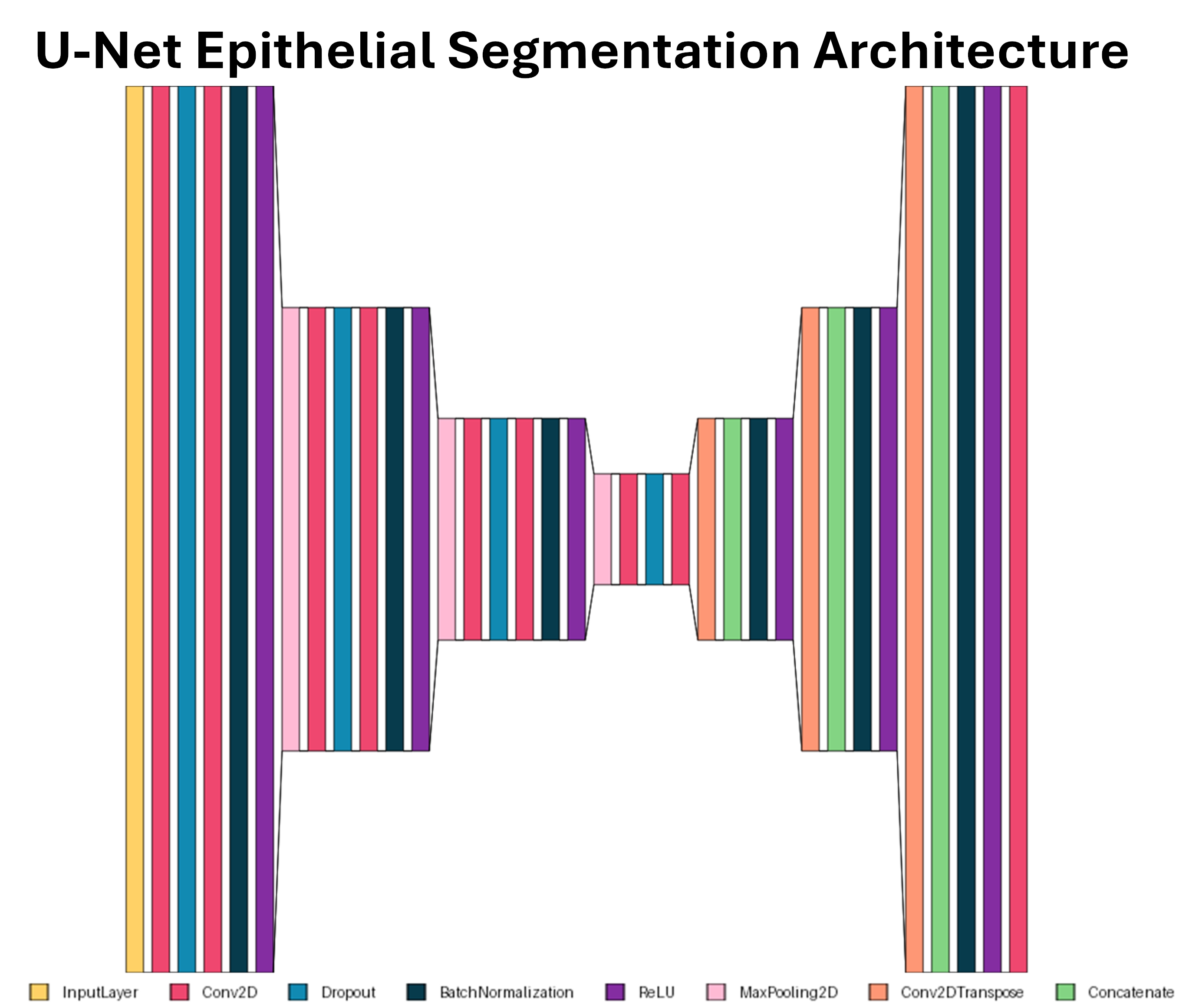

Supplement: S4 Fig — (TIFF) [file pone.0345014.s004.tiff]

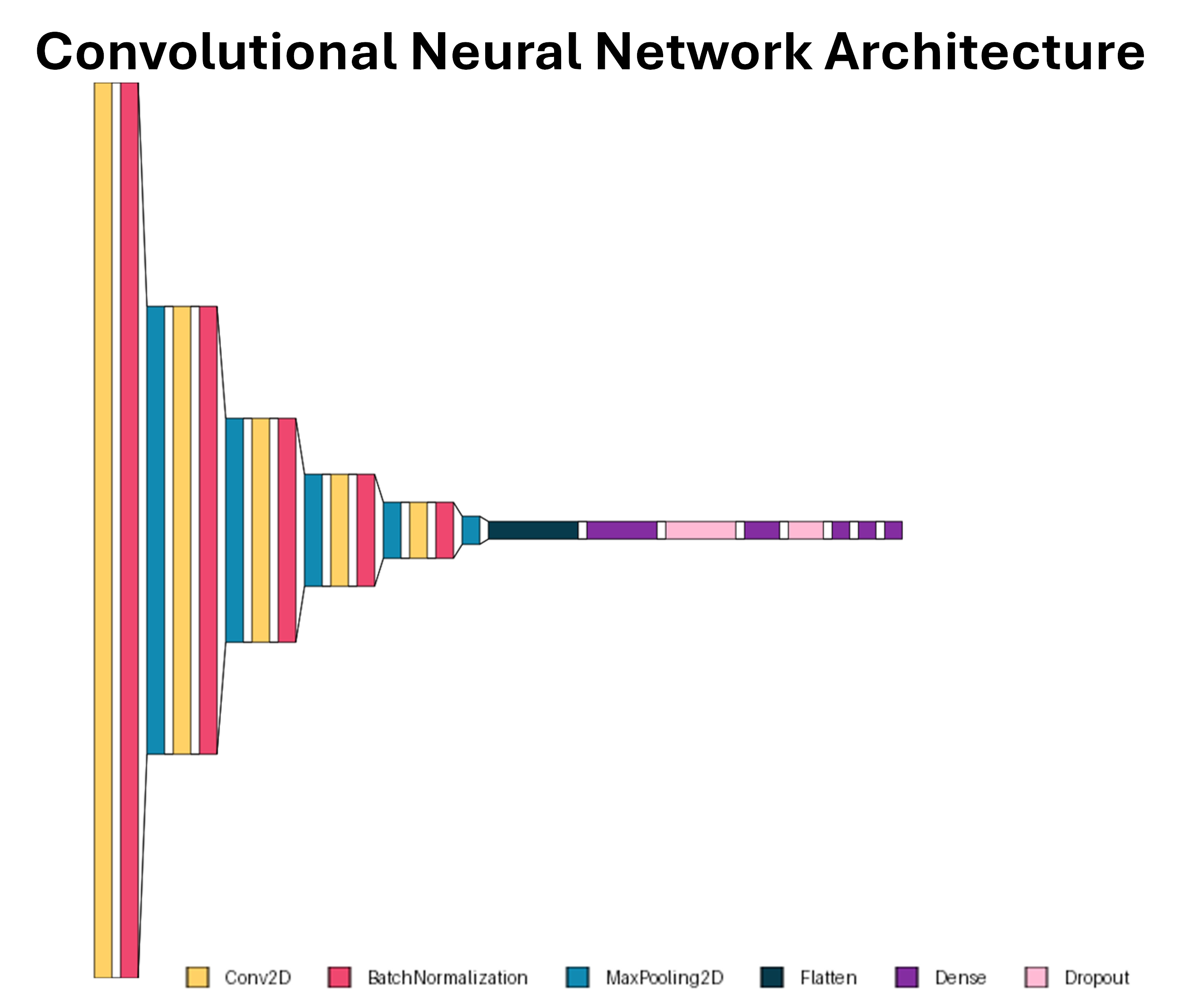

Supplement: S5 Fig — (TIFF) [file pone.0345014.s005.tiff]

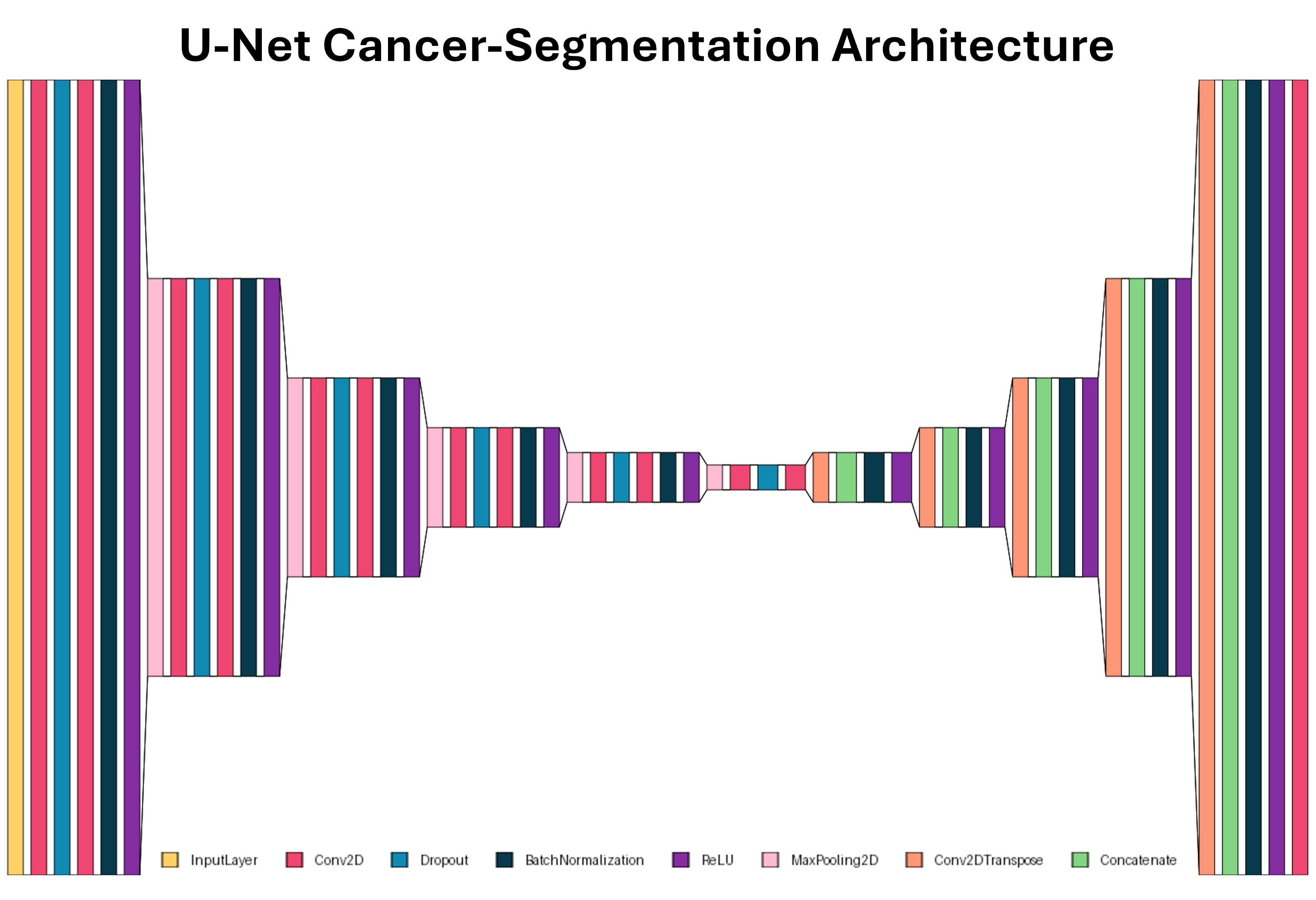

Supplement: S6 Fig — (TIFF) [file pone.0345014.s006.tiff]

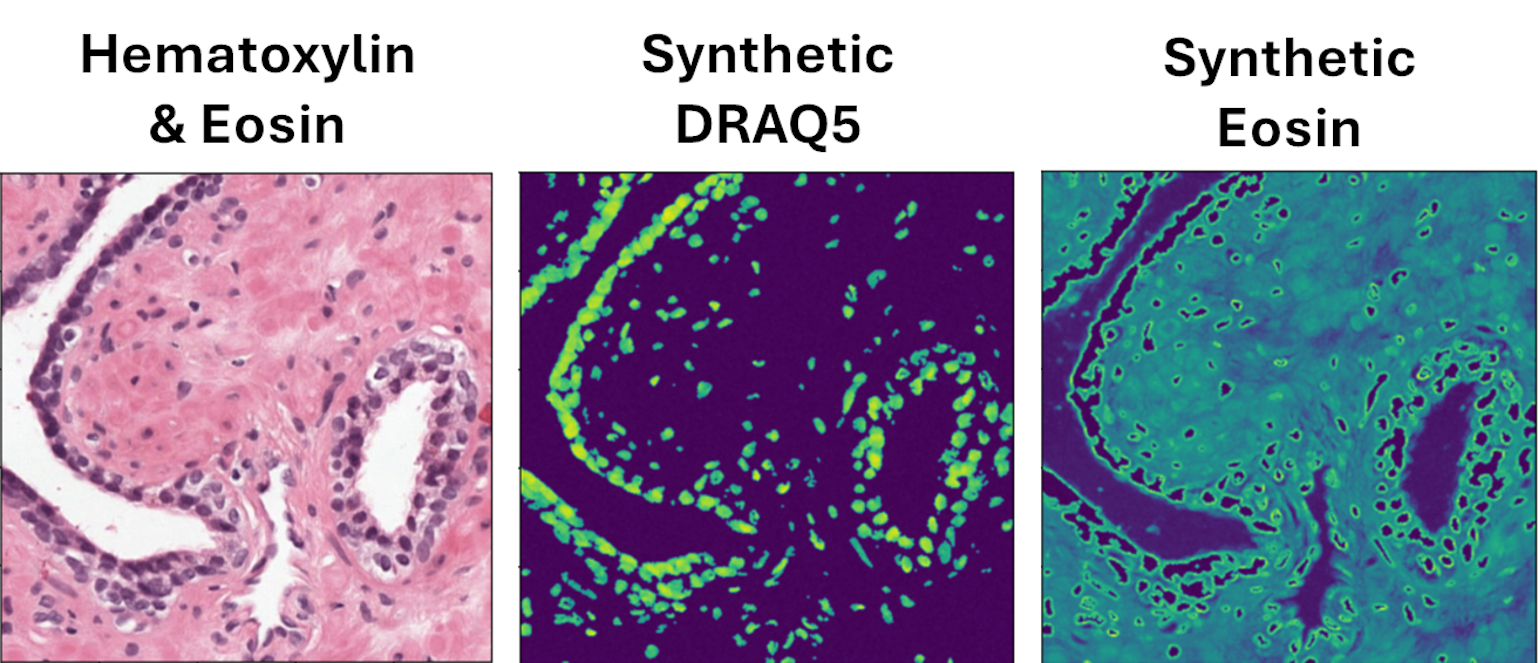

Supplement: S7 Fig — (TIFF) [file pone.0345014.s007.tiff]

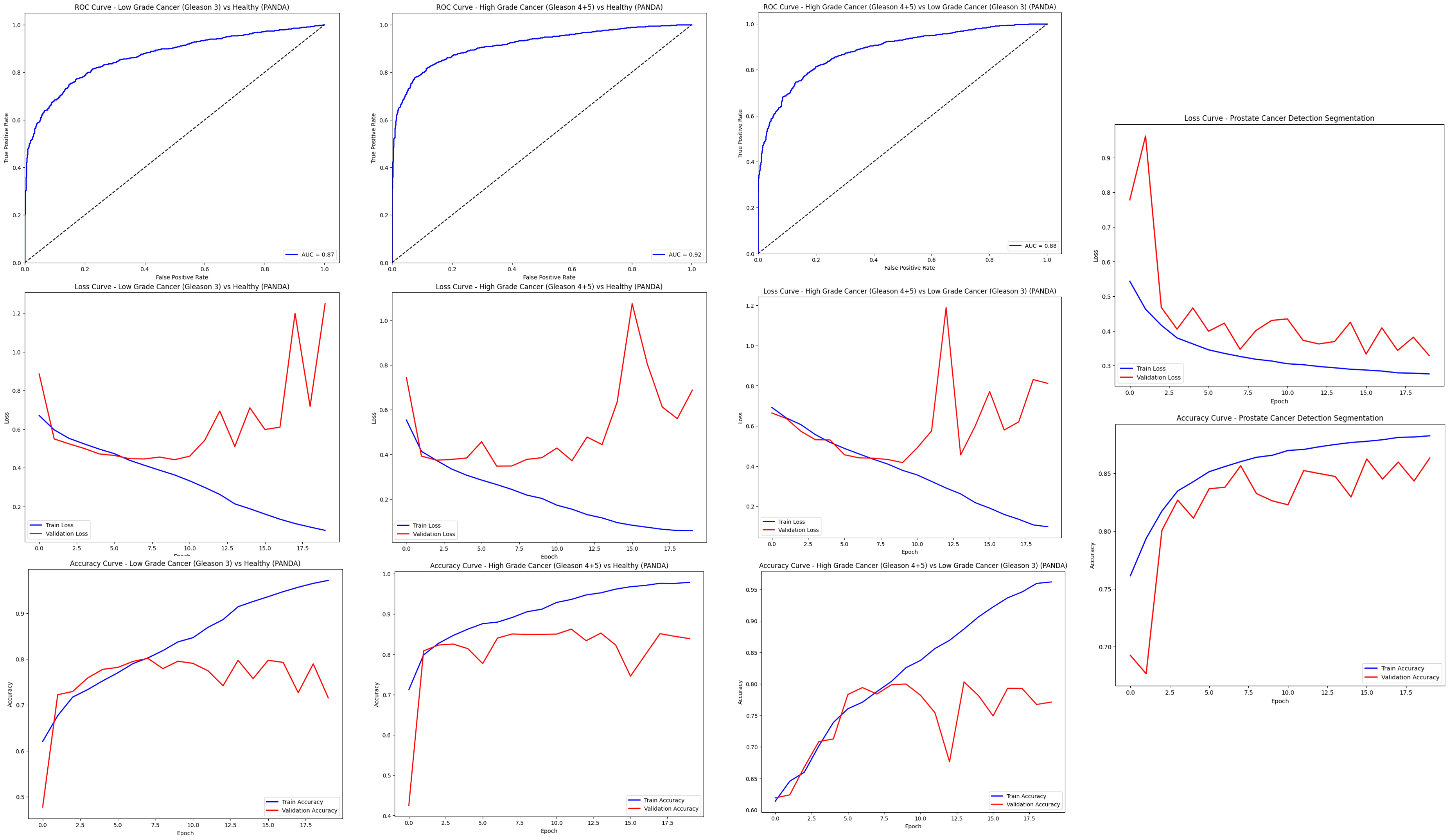

Supplement: S8 Fig — (TIFF) [file pone.0345014.s008.tiff]
