# Supplementary material for: Prostate cancer tissue mapping and stratification using DRAQ5 and Eosin fluorescent labels integrated with AI classification and segmentation algorithms
Source: PLoS One. 2026 Mar 26;21(3):e0345014. doi: 10.1371/journal.pone.0345014 (PMC13021167; doi:10.1371/journal.pone.0345014)
Supplement: S1 Table — The inter-observer metrics (retrieved after epithelial segmentation is applied to the masks) for comparing mask overlap are the DICE score (which compares background to annotated region), and the quadratic weighted Cohen Kappa score (which also considers grades assigned (Healthy, Low Grade Cancer, High Grade Cancer)). Manual Tr: Manually Transferred Annotations, Path A: Pathologist A, Path B: Pathologist B. (PDF) [file pone.0345014.s009.pdf]

Prostate Cancer Tissue Mapping and Stratification using DRAQ5 and Eosin Fluorescent Labels integrated with AI Classification and Segmentation Algorithms

Michail Georgios Papachristos[1], Emiliano Spezi[2], Carolina Fuentes[3], Ioulia Evangelou[4], David Hywel Thomas[5], Fiyinfoluwa Akinade[1], Marie Wiltshire[1], Anna Wilson[6] Rachel J Errington[1] Dimitris Parthimos[1]

1 School of Medicine, Division of Cancer and Genetics, Cardiff University, Cardiff, UK
2 School of Engineering, Cardiff University, Cardiff, UK
3 School of Computer Science and Informatics, Cardiff University, Cardiff, UK
4 Swansea Bay UHB Pathology laboratories, Morriston, Singleton, Swansea, UK
5 Department of Cellular Pathology, University Hospital of Wales, Heath Park, Cardiff, UK
6 University of Alabama at Birmingham School of Medicine, Birmingham, Alabama, USA

* Corresponding author
PapachristosMG@cardiff.ac.uk

Abstract

Background: Fluorescent microscopy using the DRAQ5 and Eosin probes has been shown in the literature to be capable of producing rapid tissue characterization through synthetic H&E-like pseudoimages, which can be potentially utilized in the clinic. This study focuses on developing deep learning models for classification and segmentation of prostate tissue labeled with DRAQ5&Eosin. The fluorophores provide highly specific features of nuclear and cytoplasmic content that allows for enhanced spatial resolution and multi-parametric analytics. The inter-dependencies of image acquisition and configuration variability on AI predictive accuracy is systematically interrogated. We are thus able to establish limits on experimental and analytical robustness in automated Gleason Grading (1-5) tissue samples of prostate cancer.

Materials and Methods: A labeling technique based on a far-red DNA probe DRAQ5, and Eosin allowed us to generate a two-channel fluorescent readout of prostatic tissue samples. Deep learning networks were employed to classify and segment DRAQ5 and Eosin fluorescent image regions into healthy and high/low grade cancerous tissue. A subset of images were acquired with variable microscopy configurations (focus, noise, zoom, lens) to evaluate the robustness of the proposed experimental-analytical pipeline and reproducibility of predictions.

Results: Machine Leaning classifiers of High Grade Cancer (Gleason pattern 4 or 5) vs Healthy, Low Grade Cancer (Gleason pattern 3) vs Healthy, and High Grade Cancer vs Low Grade Cancer achieved an area under the curve of 0.9314, 0.8398, and 0.7715 respectively. Pixel wide cancer segmentation attained DICE scores of 0.8436, 0.5138, and 0.705 for background, healthy, and cancerous tissue respectively. The segmentation model also displayed robustness against a broad range of induced acquisition variability.

Conclusion: Overall, DRAQ5 and Eosin labeling in combination with AI tools demonstrate a potential pipeline used in diagnostic clinical application when

employing fluorescent imaging. Future research could expand and bring this combined fluorescent biomarker and AI methodology to the clinic.

Table S1. Comparison of manually transferred annotations (transferred from the sequential H&E to the D&E images), to the annotations by the two expert pathologists on the corresponding synthetic H&E (which is created from the D&E) on a subset of 9 WCB TMA cores. The inter-observer metrics (retrieved after epithelial segmentation is applied to the masks) for comparing mask overlap are the DICE score (which compares background to annotated region), and the quadratic weighted Cohen Kappa score (which also considers grades assigned (Healthy, Low Grade Cancer, High Grade Cancer)). Manual Tr: Manually Transferred Annotations, Path A: Pathologist A, Path B: Pathologist B.

Comparison	Manual Tr VS Path A		Manual Tr VS Path B		Path A VS Path B	
Metric	DICE	Cohen Kappa	DICE	Cohen Kappa	DICE	Cohen Kappa
Core 1	0.961	0.964	0.981	0.977	0.96	0.965
Core 2	0.851	0.586	0.884	0.632	0.898	0.89
Core 3	0.86	0.851	0.779	0.765	0.781	0.786
Core 4	0.965	0.897	0.968	0.9	0.957	0.948
Core 5	0.868	0.857	0.937	0.931	0.866	0.854
Core 6	0.892	0.88	0.816	0.797	0.816	0.799
Core 7	0.92	0.913	0.938	0.713	0.924	0.73
Core 8	0.925	0.856	0.912	0.847	0.916	0.924
Core 9	0.884	0.819	0.921	0.89	0.833	0.813
